# A WSN and LoRa Hybrid Multimedia Transmission Protocol for Scalar Data and Image Transmission †

**DOI:** 10.3390/s24248165

**Published:** 2024-12-21

**Authors:** Quoc Hop Ta, Van Khoe Ta, Trang Tien Nguyen, Hoon Oh

**Affiliations:** 1 Ubicom Laboratory, Department of Electrical, Electronic and Computer Engineering, University of Ulsan, Ulsan 44610, Republic of Korea; mchh2113@gmail.com; 2 Faculty of Electronic Telecommunication Engineering, Le Quy Don Technical University, Hanoi 11917, Vietnam; tavankhoe@gmail.com; 3 HTWave Co., Ltd., Ulsan 44623, Republic of Korea; nttien3i@gmail.com

**Keywords:** wireless sensor networks, LoRa networks, hybrid networks, pipelined transmission, reliability, end-to-end image transmission delay

## Abstract

The proposed protocol features reliable and fast image transmission while periodically transmitting scalar data without interruption by allowing two networks, a LoRa network and a wireless sensor network, with different transmission characteristics to cooperate. It adopts the RT-LoRa protocol for periodic scalar data transmission and uses a WSN-based pipelined transmission method that leverages single-hop message transmission of a LoRa network for image transmission. Thus, it can not only eliminate the control message overhead for time synchronization, slot scheduling, and path establishment for pipelined image transmission in WSNs but also eliminate interferences within WSNs, such as data collisions and data and message collisions, during pipelined image transmission, thereby enabling high reliability and fast transmission. According to experimental results obtained inside a university building, the proposed protocol achieved an image transfer rate of approximately 96% without packet loss, transmitted one 24 KB image in approximately 0.3 s, and achieved an image transfer rate of 100% under the tolerance of one image packet loss. These results indicate a speedup of about 25% compared to a recent pipelined protocol while ensuring near-perfect image transmission quality.

## 1. Introduction

Much effort has been made to automate various systems, such as industrial process management, work safety management, worker health management, and environmental management, as well as to increase the operation speed and accuracy of such systems. In those systems, various sensor devices sense the situation and then transmit scalar data (i.e., only a few bytes or, at most, tens of bytes) to a server periodically using a data collection network such as LoRa networks (LoRaNets) [1,2], wireless sensor networks (WSNs) [3,4], or narrowband Internet of things (NBIoTs) [5,6], and the server judges the situation based on the analysis of those data, and takes actions. The recent trend is sensor devices equipped with a small camera that transmit short video or images, on demand or when needed, to a server that determines the situation using artificial intelligence (AI) image analysis technology.

Although transmitting both scalar data and images using a data collection network is of importance from a spectrum-efficient perspective, there are some technical requirements. First, the image must be transmitted quickly. Excessive transmission delays can cause serious problems, as the server may be requesting images to check for emergency situations. However, if the periodic transmission of scalar data interferes with image transmission, the image transmission delay may increase. Second, the image must be transmitted reliably since image quality determines the accuracy of judgment. Even in WSNs with relatively high data rates, the loss of image packets can increase due to the interferences of control messages and/or scalar data. A typical approach to solving this problem is to stop transmitting scalar data while transmitting an image and to minimize the number of control messages.

Recently, LoRa technology has attracted attention in industrial applications due to its long transmission range and high link stability with a simple star topology [7]. However, LoRa technology has a low data rate, making it difficult to transmit images [8]. On the other hand, WSN has much higher data rates than the LoRa network but may require time synchronization and slot scheduling, and it also uses control messages frequently to transmit images reliably over multiple hops. Therefore, the shortcomings of these individual networks motivated us to design a hybrid protocol that leverages the strengths of both WSN and LoRaNet.

Although much research has been performed to improve the transmission delay and/or stability of image transmission in WSN or LoRa networks, there has been little research on image transmission based on hybrid networks. In terms of network architecture, these studies are categorized into three types: WSN only, LoRaNet only, and WSN and LoRa hybrid network (HybridNet). In [9], the authors tried to avoid data collisions by allocating nodes with different time slots and combining information from routing and MAC layers, thereby enabling more packet movements. The authors in [10] proposed an intelligent, secured two-way image transmission protocol using the Corvus Corone module over WSN. However, those protocols do not address image transmission delay. Some protocols use slot scheduling and pipelined transmission to avoid message or data collisions [11,12]. The authors in [11] proposed the efficient multipath pipeline transmission (EMP) protocol to improve transmission reliability by recovering lost packets using both hop-by-hop and path-wide retransmissions and to reduce image transmission delay using multipath pipelined transmission with multiple channels. This approach suffers from overhead time synchronization, slot scheduling, and path establishment. The authors in [12] proposed a pipelined cooperative transmission (PCT) protocol based on a cooperative path where all path nodes on the image transmission path have their respective cooperative nodes. Then, if a node fails to transmit a packet, the cooperating node of its downstream node that overhears the packet forwards it to the next downstream node, thus achieving fast forwarding; the protocol uses the acknowledgment message to enable fast forwarding.

Meanwhile, some researchers tried to overcome the limitations of LoRaNet by reducing unnecessary packet transmissions [13,14] or reducing the image size with encryption techniques [15]. The authors in [13] improved image transmission speed in LoRaNet by collectively acknowledging multiple packets, reducing unnecessary traffic, and enhancing throughput with a protocol that reserves channels between gateways and nodes to prevent collisions when multiple nodes transmit images. In paper [14], the sender transmits image packets continuously upon receiving a request in each communication round, and the receiver requests retransmissions for any lost packets after receiving the end flag. The authors in [15] demonstrated point-to-point image transmission over the LoRa physical layer, where images are encrypted and divided into packets for transmission. However, these methods are limited by LoRa’s low data rate, justifying the need for a hybrid approach that combines the strengths of both WSN and LoRaNet.

Researchers in [16,17,18,19] improved network performance by combining the high-speed and short-range capabilities of WSN with the long-range and low data rate capabilities of LoRaNet. In [16], the authors presented a fire detection and evacuation model using LoRaNet and ZigBee, where two communication technologies with different transmission ranges and data rates cooperate to transmit sensor and visual data for quick fire detection and evacuation optimization. The authors in [17] proposed a HybridNet layered model that combines ZigBee and LoRa to reduce deployment costs and increase network lifetime for photovoltaic monitoring. In [18], the authors evaluated a heterogeneous architecture combining LoRaNet and WSN in terms of energy-efficient data collection in large-scale networks. The authors of [19] developed a hybrid LoRa-ZigBee device that can utilize long-range LoRa communication to overcome the inefficiency of ZigBee in transmitting control commands and managing the network in wireless mesh networks. However, none of these papers addressed the stability or delay issues of image transmission.

This paper discusses a hybrid multimedia transmission (HMT) protocol, an extension of the conference paper [20], that enables periodic transmission of scalar data and reliable fast transmission of images. The HMT protocol uses LoRaNet to transmit scalar data and control messages, such as a time synchronization message for the start of image transmission, a WSN path establishment message for image transmission, and a retransmission request message to salvage missing packets while it solely uses WSN to transmit image packets in a pipelined manner. Thus, the HMT protocol can eliminate the WSN control overhead that occurs when using only WSN and allow all nodes to transmit scalar data without interruption, regardless of image transmission. In addition, the HMT protocol can not only transmit images quickly using WSN but also achieve extremely high reliability by simply retransmitting only a few missing image packets that are notified using LoRaNet by GW.

The rest of this paper is organized as follows. Section 2 defines the network model and explains the motivation of this study. In Section 3, the proposed approach is formally described and followed by a performance evaluation in Section 4. Finally, concluding remarks are provided in Section 5.

## 2. Preliminaries

### 2.1. Network Model

The considered hybrid network consists of one gateway (GW) acting as both WSN sink and LoRa gateway, a number of end nodes acting as scalar nodes (SNs), and some end nodes acting as both scalar and multimedia nodes (MNs). Every end node has both an IEEE 802.15.4 transceiver and a LoRa transceiver. Thus, the hybrid network can act as a wireless sensor network (WSN) and a LoRa network. Both SN and MN can transmit scalar data at regular intervals, while MN can additionally transmit an image captured from a camera at the same time. GW is wall-powered, and both SN and MN are battery-powered. All end nodes send (receive) small data directly to (from) the GW using LoRaNet, and some of them can transmit an image according to the image transmission path on WSN (WSN path or *WSNpath*) to GW.

**Figure 1:**
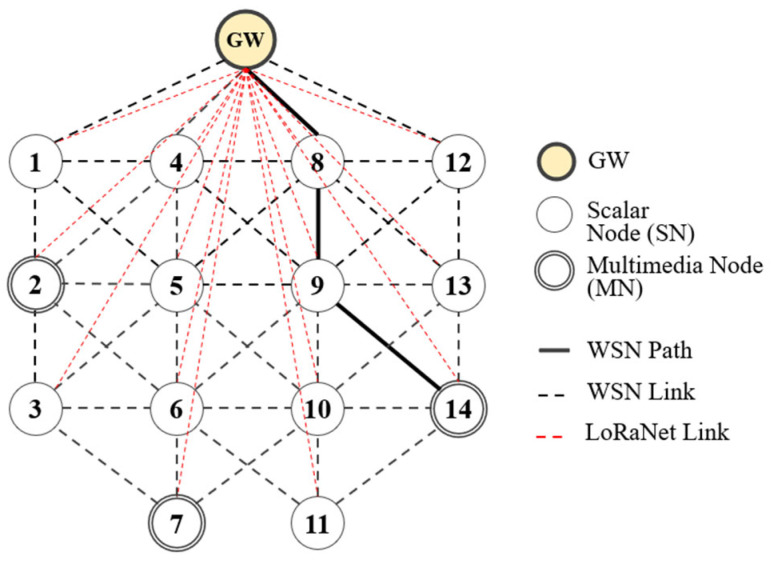
HybridNet model.

Figure 1 shows a HybridNet model with one GW, 11 SNs, and 3 MNs. The black dashed lines and the red dashed lines indicate WSN and LoRaNet links, respectively. The thick solid line indicates *WSNpath* along which node 14 can send an image to GW.

### 2.2. Motivation

There are a variety of wireless communication technologies that can be used to build industrial data collection networks, but among them, WSN and LoRaNet are receiving much attention. WSNs have relatively high data rates (up to 250 kbps), but their short transmission range requires multi-hop routing for data transmission. Such multi-hop data transmission is accompanied by high overhead due to control messages to perform tasks such as topology management, route establishment, slot scheduling, and time synchronization for reliable data transmission. LoRaNet, on the other hand, has very low data rates (up to 5470 bps for SF7) but offers a long transmission range and high link reliability against various wireless communication obstacles. However, it suffers from high data collision probability due to long Time on Air (ToA), and this phenomenon becomes more severe as traffic increases.

The types of data typically transmitted using wireless data collection networks in industrial environments are scalar data ranging from a few bytes to hundreds of bytes and images ranging from a minimum of 100 × 80 pixels to a maximum of 512 × 512 pixels. These scalar data are primarily collected periodically, whereas images are collected on an as-needed basis. For example, consider a fire detection system for the safety of workers working in hazardous areas. The server detects signs of fire by periodically collecting and analyzing scalar data such as temperature, oxygen concentration, toxic gas, and smoke from sensor nodes installed in the area. If a fire is suspected, the server can confirm the situation by requesting images from camera sensors installed at the target location.

The slot scheduling approach in LoRaNet provided in [21] can be employed for the periodic collection of scalar data. However, LoRaNet is not suitable for transmitting images due to its low data rate. For example, one packet of a 37-byte payload (excluding the MAC header of 10 bytes) can be transmitted every 100 ms with the spreading factor 7 (SF7) [22] in LoRaNet. If the 512 × 512-pixel RGB image is compressed using the JPEG2000 compression method with a compression ratio of 1:33.65, the image size of 786,432 bytes is reduced to approximately 23,400 bytes, resulting in 64 packets. Then, it takes about 64 s to transmit one image. Thus, it is necessary to consider WSNs with a high data rate. However, when using only WSN, not only is it difficult to periodically and reliably transmit scalar data through multi-hop, but scalar data transmission must be temporarily stopped during emergency image transmission to prevent transmission interference.

In hybrid networks, LoRaNet can be used for periodic scalar data transmission, topology management, WSN path establishment for image transmission, and control message transmission, while WSN is used for image transmission on demand. In WSN, one packet (maximum payload of 110 bytes excluding MAC header) can be transmitted in less than 5 ms per hop, so it can be transmitted up to a distance of 10 hops within 50 ms. This implies that even if one packet is transmitted at 50 ms intervals to avoid collision, one compressed image of 23,400 bytes (≈213 packets) can be transmitted in approximately 10.65 s. This transmission delay can be dramatically reduced if pipeline transmission is applied on a multi-hop path.

### 2.3. Overview of Pipelined Transmission

**Figure 2:**
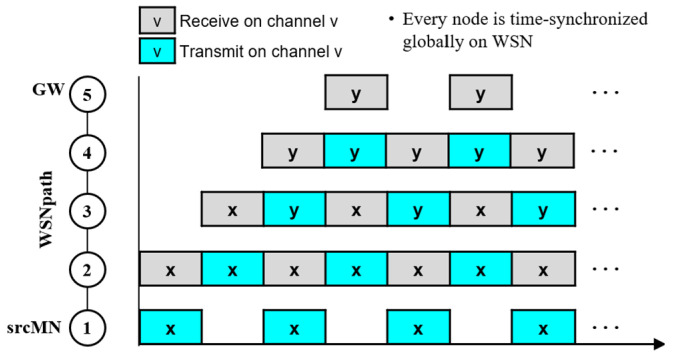
Pipelined image transmission using 3 channels.

Referring to Figure 2, let us explain how an image is transmitted from 1 to 5 in a pipelined manner, where the WSN path is (1, 2, 3, 4, 5). Assuming that all nodes are globally time-synchronized, each node schedules its time by dividing it into a series of transmit and receive slots. Then, all intermediate nodes (2 to 5) alternate between receiving and transmitting, starting from the receiving slot where they receive the first packet. Node 1, which transmits the image, transmits the next packet every two slots from the time it transmits the first packet. This is because LoRa nodes operate in half-duplex mode.

In this case, collisions may occur between packets transmitted by different nodes in the same slot. Multiple channels can be used to prevent collisions. For example, nodes 1 and 3 (or 2 and 4) can transmit packets simultaneously using three different channels *x* and *y*, respectively. The number of channels used can be limited by using the spatial slot reuse technique [23].

HybridNet allows us to improve the above pipelined transmission method without time synchronization and slot scheduling. Suppose that GW requests image transmission from a specific MN (or source MN) by broadcasting an *image request message* (*IREQ*) using LoRaNet. If GW maintains a whole WSN topology, it can calculate *WSNpath* from *srcMN* to GW based on the WSN topology and then include the *WSNpath* and the ID of *srcMN* in *IREQ*. Upon receiving *IREQ*, *srcMN* starts transmitting image packets immediately, and other nodes on the path wait to receive an image packet. Upon receiving an image packet, a node on the path changes its channel to the channel of its downstream node and forwards the received packet to its downstream node. At completion, the node changes its channel back to the previous channel, waiting for the next packet from its upstream node. This approach simplifies the implementation of pipelined transmission by eliminating the need for time synchronization and slot scheduling.

### 2.4. Notations and Messages

Some notations and messages are summarized in Table 1 and Table 2, respectively.

## 3. Hybrid Multimedia Transmission Protocol

### 3.1. Protocol Structure

**Figure 3:**
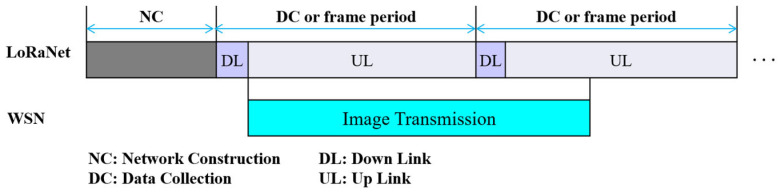
Protocol structure.

The protocol structure starts with network construction (NC) and repeats the data collection (DC) period, as shown in Figure 3. The DC period is divided into a downlink period (DL) and an uplink period (UL), and image transmission is initiated by the request of a server.

During NC, every node registers with the server using LoRaNet and manages its neighbors (or the WSN link state) by exchanging a hello message (*Hello*) with its neighbors using WSN. At the beginning of every DL period, GW broadcasts a downlink message using LoRaNet, which may include an image request command to request an image from a specific multimedia node. Upon receiving the image request command, image transmission is immediately performed on WSN while every node transmits scalar data to GW during the UL period.

### 3.2. Protocol Operation

The proposed protocol uses two LoRa channels, *dCH* and *dCHm*, where the former is a common data channel used by all nodes for communication during DL and UL periods, and the latter is a data channel exclusively assigned to a source multimedia node (*srcMN*) and GW during image transmission.

At the beginning of DL, GW transmits *DLm* = ([*IREQ*]) using *dCH*, where *IREQ* is an image request command:

*IREQ* = (*srcMN*, *WSNpath*, *WSNChList*, *dCHm*)

where *WSNpath* indicates an *image transmission path* from *srcMN* to GW, and *WSNChList* is the list of WSN channels for pipelined image transmission. Upon receiving *DLm*, every node wakes up its WSN module, but only every *k* frame period, where *k* is an integer, and broadcasts *Hello* to its neighboring nodes after a random delay. Then, every node, say *x*, can update its link state, *NS*[*x*], represented as the set of its neighbors.

The proposed protocol employs RT-LoRa [21] to transmit scalar data. In RT-LoRa, every node registers with a server via GW by sending its profile, including its ID, data transmission period, and WSN link state during the NC period using LoRaNet. Thus, a server can maintain the profiles of all participating nodes and the whole WSN topology and then share the profiles with all nodes. Each node then generates a slot schedule based on the profiles of all nodes, thereby avoiding collisions between data transmissions from different nodes. For a detailed description of slot scheduling, see paper [21]. According to its slot schedule, each node transmits an uplink message to GW:

*ULm* = (*x*, *scalarData*, [*NS*[x]])

where *x* indicates node ID and *scalarData* indicates the scalar data to be sent.

If GW needs an image from any MN, say *srcMN*, it calculates *WSNpath* based on the WSN topology and broadcasts an *image request message*, *IREQ* = (*srcMN*, *WSNpath*, *WSNChList*, *ch*) using *dCH*, where *WSNChList* is the list of WSN channels used in pipelined image transmission, and *ch* indicates *dCHm*. Upon receiving *IREQ*, all nodes on *WSNpath* activate their WSN modules, and *srcMN* transmits image packets (*iPackets*) along the WSN path.

**Figure 4:**
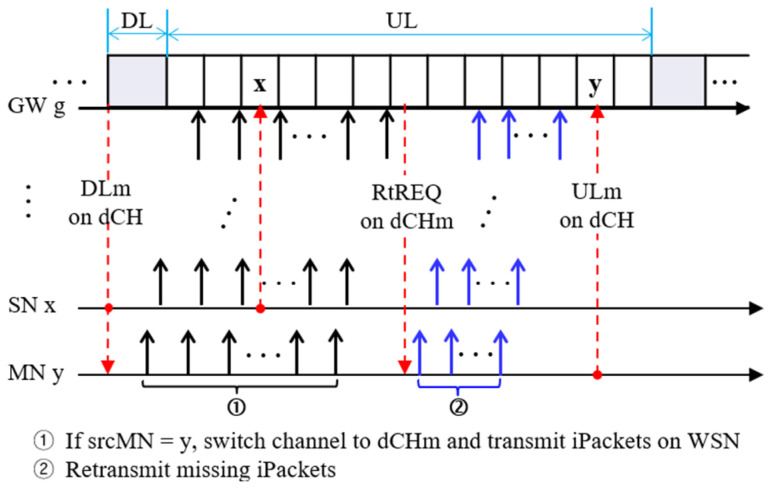
The protocol operation in a hybrid network.

Figure 4 illustrates protocol operation where GW broadcasts *DLm* during the DL period, and nodes *x* and *y* send *ULm*, including scalar data according to the slot schedule during each UL period using LoRaNet. Upon receiving *DLm* = (*IREQ*), all nodes on *WSNpath* activate their WSN modules. Suppose that *WSNpath* = (*y*, *x*, …, *g*). Then, *srcMN y* switches its channel to dCHm and starts image transmission along *WSNpath*. When node *y* completes the transmission of all *iPackets*, GW *g* transmits a *retransmission request message*, *RtREQ* = (*m*_1_, …, *m_k_*), where *m_i_*, *i* = 1… *k*, indicates all dropped packets during the first round of image transmission on channel *dCHm*. Upon receiving *RtREQ*, *srcMN* starts the second round to transmit missing iPackets. If the maximum number of retransmissions (*MaxRTs*) is *unity*, *srcMN* changes its channel back to *dCH*.

As explained above, scalar data can be transmitted without interruption according to the slot schedule even while transmitting images. In this way, the two different networks maximize their respective spectral efficiencies.

### 3.3. Scalar Data Trasmission on LoRaNet

#### 3.3.1. Slot Scheduling and Scalar Data Transmission

Assume that the UL period is divided into *2^N^* slots such that each slot is large enough to send one scalar datum. Each node states its transmission period as a length of $2^{k}$ slots where 0 ≤ *k* ≤ *N*. For example, if a node specifies *k* = *N*, it means that it transmits one scalar datum within one UL period. Then, node *x* transmits the following profile information *NPI*(*x*) to GW during the NC period for registration:

$$NPI\;\left(x\right)=\left(x,\;k\right)$$

Then, the slot demand *SD*(*x*) of node *x* within one UL period is given as follows:

(1) $$SD\;\left(x\right)=\;2^{N-k}$$

Given *n* participating nodes, GW manages a node profile information (*NPI*) for the whole nodes as follows:

$$NPI=\left(PI\left(1\right),\;PI\left(2\right),\;\ldots ,\;PI\left(n\right)\right)$$

If GW distributes *NPI* to all nodes, every node can generate the identical slot schedule in a distributed manner by utilizing the logical slot indexing (*LSI*) algorithm [21] that assigns a logical slot index to each of the slots in the UL period to make slot scheduling easy.

**Figure 5:**
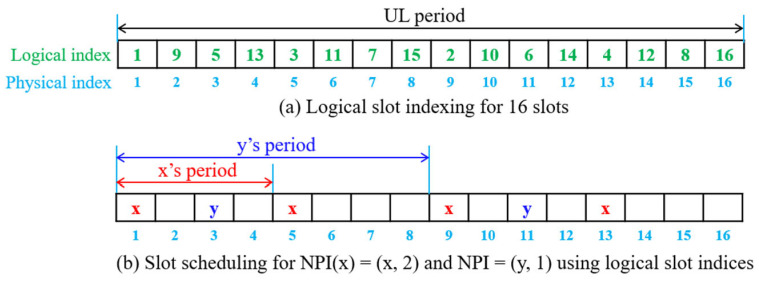
Example of logical slot indexing and slot scheduling.

Figure 5a shows an example of logical slot indices assigned by the LSI algorithm for one UL period with 16 slots. Then, the slot scheduling for scalar data transmission is explained as follows. Given the UL period with logical slot indices and *NPI*, node *i* is assigned *SD*(*i*) slots sequentially starting with the start logical slot index of node *i*, *startLSI*(*i*):

(2) $$startLSI\left(i\right)=\left\{\begin{array}{c} 1,\;\;i=1\\ startLSI\left(1\right)+\overset{i-1}{\underset{j=1}{\sum }}SD\left(j\right),\;\;i>1 \end{array}\right.$$

Let us see an example of a slot scheduling for *NPI* = (*NPI*(*x*), *NPI*(*y*)) where *NPI*(*x*) = (*x*, 2) and *NPI*(*y*) = (*y*, 1), and *SD*(*x*) = 4, and *SD* (*y*) = 2. Since node *x* has *startLSI*(*x*) = 1, it takes logical slots, 1, 2, 3, and 4 (physical indices 1, 5, 9, and 13). Then, node *y* has *startLSI*(*y*) = 5, taking two logical slots, 5 and 6 (physical indices 3 and 11). The slot schedule is provided in Figure 5b, where nodes *x* and *y* transmit one packet every four slots and every eight slots, respectively.

#### 3.3.2. Time Synchronization on LoRaNet

**Figure 6:**
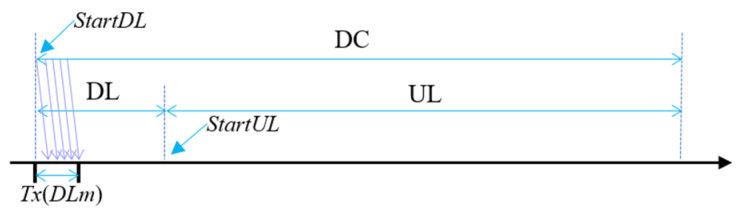
Calculating the start time of the UL period.

Let us first calculate the start time of the UL period, *StartUL*. GW broadcasts *DLm* at the beginning of the DC period, *StartDL*. Upon receiving *DLm*, every end node calculates the start time of the UL period, *StartUL*, as illustrated in Figure 6:

(3) $$StartUL=sysTime-T_{x}\left(DLm\right)+DL$$

where *sysTime* is the local time at which the corresponding node finishes receiving *DLm* and *Tx*(*DLm*) indicates the transmission time of *DLm*. Then, every node obtains its transmission slots for scalar data transmission.

### 3.4. Pipelined Image Transmission on WSNpath

**Figure 7:**
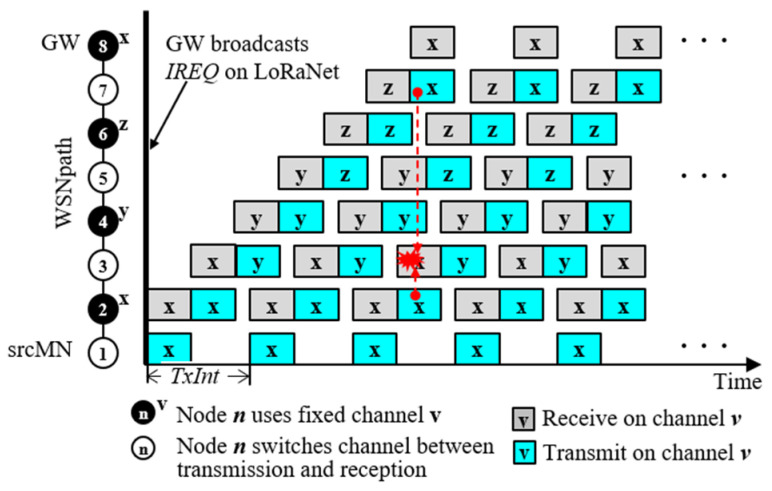
Pipelined image transmission on WSNpath from srcMN to GW.

HMT uses three channels, *x*, *y*, and *z,* for pipelined transmission. Given *WSNpath* = $\left(N_{1},N_{2},\ldots ,N_{m}\right)$, channel assignment for the nodes on the path is as follows:

(4) $$\begin{array}{l} \;\;\;\;For\;k=0,\;1,\;2,\ldots ,\\ \;\;\;\;\left(N_{6k+2}.ch,N_{6k+4}.ch,N_{6k+6}.ch\right)=\left(x,\;\;y,\;\;z\right),\\ \;\;\;\;\left(N_{6k+3}.rch,N_{6k+5}.rch,N_{6k+7}.rch\right)=\left(N_{6k+2}.ch,N_{6k+4}.ch,N_{6k+6}.ch\right),\\ \left(N_{6k+3}.tch,N_{6k+5}.tch,N_{6k+7}.tch\right)=\left(N_{6k+4}.ch,N_{6k+6}.ch,N_{6k+2}.ch\right),\\ \mathrm{and}\;srcMN.tch=N_{2}.ch \end{array}$$

where *v.ch* indicates a channel dedicated to node *v*, and *v.rch* and *v.tch* indicate a reception channel and a transmission channel that node *v* has to use for receiving and sending data, respectively. In Figure 7, the first three even-numbered nodes, 2, 4, and 6, on *WSNpath* = (1, 2, 3, 4, …, 8) are fixedly assigned channels *x*, *y*, and *z*, respectively, and odd nodes (3, 5, 7, …) continuously switch between the upstream node channel to receive a packet and the downstream node channel to transmit a packet.

Let us see how pipelined image transmission works. When every node receives *DLm* of *IREQ* = (*srcMN*, *WSNpath*, WSNChList, *dCHm*) on the common channel, *dCH*, using LoRaNet, it wakes up its WSN module if it is on *WSNpath*. Nodes except for srcMN wait for *iPacket* on its reception channel using WSN, while *srcMN* transmits *iPacket* at regular intervals. Since LoRa works as a half-duplex mode, *TxInt* as the interval of two consecutive packets transmitted by *srcMN* can be calculated as follows:

(5) $$TxInt\;\geq \;2\times T_{h}+\alpha$$

where $T_{h}$ is one-hop transmission delay of one packet and α is the channel switching delay. Upon receiving *iPacket*, every intermediate node immediately transmits the packet on the transmission channel and then changes its channel back to its reception channel. If a signal transmitted from one node reaches another node using the same channel that is four hops away, collision may occur, as shown by the red dotted line in Figure 7. Thus, we assume that the signal interference range is less than four hops. In this way, *iPackets* are transmitted at *TxInt* intervals without time synchronization and slot scheduling.

The pipelined image transmission algorithm for WSN is detailed in Algorithm 1. The algorithm is presented in the form of a simple network program that defines the behavior of *srcMN* and intermediate nodes. All statements except for the *chMapping*() function in line 22 have O(1) complexity, and *findPos*() in line 23 is executed only once, and its complexity is O(*h*) because it uses a linear search where *h* is the length of *WSNpath*.
**Algorithm 1.** Image transmission algorithm.//P(v): v’s downstream node //C(v): v’s upstream node //v.rch: v’s reception channel //v.tch: v’s transmission channel1    At GW://requests image from srcMN 2       broadcast IREQ = (srcMN, WSNpath, WSNChList, dCHm); 3    At node v that receives IREQ: 4    if v = srcMN then 5       curTime = systemTime(); 6       set nextTxTimer = curTime + TxInt; 7       change myLoRaCh to dCHm;//channel switching 8       srcMN.tch = WSNChList [0];//takes the first channel 9       transmit iPacket on srcMN.tch using WSN; 10   else if v belongs to WSNpath then 11     turn on WSNmodule; 12     (v.rch, v.tch) = chMapping(v, WSNpath, WSNChList); 13     wait for iPacket on v.rch; 14   endif; //Transmits iPackets at TxInt intervals 15   At srcMN that nextTxTimer expires: 16     curTime = systemTime(); 17     set nextTxTimer = curTime + TxInt; 18     transmit iPacket on v.tch; //Forwarding packet and waiting for new packet 19   At node v that receives iPacket from C(v): 20   transmit iPacket to P(v) on v.tch; 21   wait for iPacket on v.rch; //myPos = index in WSNpath starting with 1 //WSNChList = list of three channels 22   function chMapping(v, WSNpath, WSNChList); //myPos > 1 since v cannot be srcMN 23     myPos = findPos(v, WSNpath); 24     if myPos is even then//dedicated channel 25          k = (myPos/2 - 1) % 3; 26          v.rch = v.tch = WSNChList [k]; 27     else//if odd 28          k = ((myPos − 1)/2 − 1) % 3; 29          v.rch = WSNChList[k];//upstream node ch 30          k = ((myPos + 1)/2 − 1) % 3; 31          v.tch = WSNChList [k];//downstream node ch 32     endif; 33     return (v.tch, v.rch); 34   endFunction

### 3.5. Analysis of Image Transmission Delay

#### 3.5.1. Packet Transmission Interval

One-hop transmission delay of one packet, $T_{h}$, is given as follows [24]:

(6) $$T_{h}=t_{mr}\left(p\right)+t_{turnon}+t_{CCA}+t_{ppd}+t_{Tx}\left(p\right)+t_{rm}\left(p\right)$$

where $t_{mr}\left(p\right)$ is the transfer time of packet p from MCU to a radio chip buffer at a sender, $t_{turnon}$ is the delay in turning on the radio chip, $t_{CCA}$ is the clear channel assessment time (8 symbols), $t_{ppd}$ is the physical layer processing delay (12 symbols), $t_{Tx}\left(p\right)$ is the time to transmit packet p, and $t_{rm}\left(p\right)$ is the time to transfer packet p from a radio chip to MCU at a receiver. The propagation delay of the packet is considered negligible compared to others. According to the IEEE 802.15.4 physical layer standard [25], since the transmission rate is at 250 kbps, $t_{Tx}\left(p\right)$ can be calculated as follows:

(7) $$t_{Tx}\left(p\right)=0.3125\;\mathrm{ms}/\mathrm{byte}\times size\left(packet\;p\right)$$

where *size*(packet p) indicates the size of packet p. If the payload is 100 bytes, size(packet p) is 105 bytes with a header of 5 bytes. In addition, $t_{CCA}=0.128\;\mathrm{ms}$, $t_{ppd}=0.192\;\mathrm{ms}$, $t_{turnon}=1.015\;\mathrm{ms}$, and both $t_{mr}$ and $t_{rm}$ are considered negligible. Then, $T_{h}\approx 4.62\;\mathrm{ms}$, according to (6), and from (5), *TxInt* is given as follows:

(8) $$TxInt\approx 9.24+\alpha \;\left(\mathrm{ms}\right)$$

Some nodes have to perform channel switching twice, once to send a packet and once to receive a packet. Since $t_{turnon}$, takes about 1.015 ms, TxInt can be modified as follows:

(9) $$TxInt\approx 11.27\;\left(\mathrm{ms}\right)$$

#### 3.5.2. End-to-End Packet Transmission Delay

Let *E2ED* be the delay time it takes for packet p to travel from source to destination. Then, it can be simply calculated as follows:

(10) $$E2ED=h\times T_{h}$$

where h indicates the number of hops between source and destination. Then, $E2ED\left(n\right)$, as the transmission delay for transmitting *n* packets from source to destination in a pipelined manner, can be calculated as follows:

(11) $$E2ED\left(n\right)=E2ED\left(1\right)+\left(n-1\right)\times \left(2\times T_{h}\right)$$

#### 3.5.3. Analysis of Image Transmission Delay

An image end-to-end delay (*imgE2ED*) is defined as the difference between the time *srcMN* starts transmitting the first *iPacket* and the time GW stops receiving *iPacket* for the same image. For convenience, *imgE2ED* for protocol *X* is denoted as $imgE2ED\left(X\right)$.

Since HMT allows retransmission for missing packets, the image transmission delay has to take into account the additional delay to retransmit the missing packets. This additional delay includes the time that GW needs to send *RtREQ* to *srcMN* and the time that *srcMN* retransmits all missing packets. Then, assuming that one image is divided into *n* image packets and *k* packets out of *n* packets are retransmitted, $imgE2ED\left(HMT\right)$ can be calculated as follows:

(12) $$imgE2ED\left(HMT\right)=E2ED\left(n\right)+E2ED\left(k\right)+T_{RtREQ}$$

where $T_{RtREQ}$ represents the elapsed time from when GW broadcasts *RtREQ* to when *srcMN* receives the command. According to [26], $T_{RtREQ}$ is expressed as the sum of preamble and payload transmission times as follows:

(13) $$T_{RtREQ}=[n_{preamble}+4.25+n_{RtREQ}]\times T_{symbol}$$

where $n_{preamble}$ is the number of symbols (=8 symbols) in the programmed preamble, $n_{RtREQ}$ is the number of symbols for *RtREQ*, and $T_{symbol}$ is the duration of one symbol. $n_{RtREQ}$ can be calculated as follows [26]:

(14) $$n_{RtREQ}=8+\max\left(ceil\left(\frac{8PL-4SF+28+16-20H}{4\left(SF-2DE\right)}\right)\left(CR+4\right),\;0\right)$$

where *SF* is the spreading factor, *PL* indicates the number of bytes in the payload, *H* determines whether the header is enabled or disabled (0: disabled, 1: enabled), *DE* determines the low data rate optimization mode (0: enabled, 1: disabled), and *CR* is the coding rate (from 1 to 4). If *RtREQ* includes *k* sequence numbers, the payload will be *k* + 1 bytes with the *srcMN* node number of one byte. Then, with *SF* = 7, *PL* = *k* + 1, *H* = 0, *DE* = 1, and *CR* = 1, $n_{RtREQ}$ can be calculated as follows:

(15) $$n_{RtREQ}=8+max\left(ceil\left(2k+6\right),0\right)$$

Moreover, the symbol duration $T_{symbol}$ is given as follows [26]:

(16) $$T_{symbol}=\frac{2^{SF}}{BW}\;\;\left(\mathrm{ms}\right)$$

where *BW* indicates bandwidth in LoRa networks. Given *SF* = 7 and BW = 125 KHz, $T_{symbol}=1.024$ ms. Assuming that *k* packets are missing out of *n* packets, according to (15), $imgE2ED\left(HMT\right)\;\left(\mathrm{ms}\right)$ can be calculated as follows:

(17) $$imgE2ED\left(HMT\right)=9.24n+11.29k+9.24H+8.4$$

## 4. Performance Evaluation

### 4.1. Scalar Data Transmission

For scalar data transmission, the HMT protocol uses the RT-LoRa protocol [21], which is briefly described in III.C. The authors evaluated the performance of RT-LoRa inside a university building using the parameters and values in Table 3. They performed experiments by varying the transmission interval from 1.5 to 12 s while each node is transmitting a 33-byte packet per transmission interval. According to experimental results, RT-LoRa achieved a transmission success rate of almost 100%.

This paper does not discuss scalar data transmission anymore. For more information regarding RT-LoRa, refer to paper [21].

### 4.2. Image Transmission

#### 4.2.1. Experiment Setup and Scenarios

For the experiment, we developed a hybrid GW in our lab, which consists of a GW mainboard, a LoRa communication module, a WSN device, and a hybrid node that has both LoRa and WSN devices interconnected by RS-232. Their specifications are summarized in Table 4.

**Figure 8:**
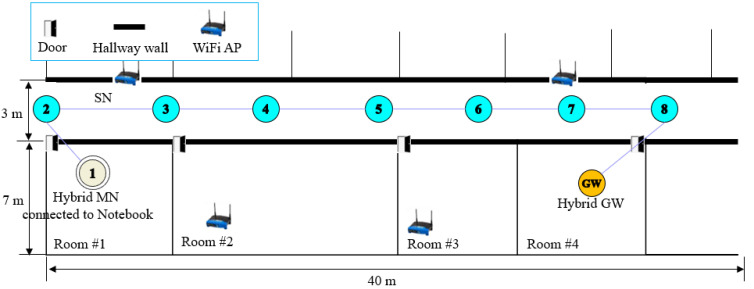
The testbed of HybridNet that consists of one GW, seven SNs, and one MN.

**Figure 9:**
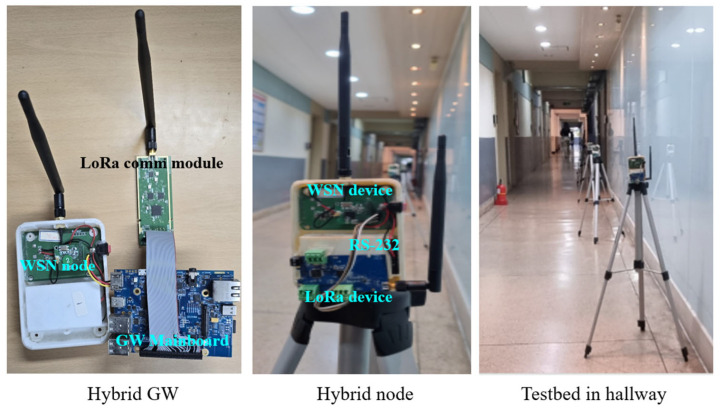
Hybrid GW, WSN node, and Testbed.

A mesh network could be built using more nodes, but scalar data transmission using LoRaNet was performed in the paper [21] and was proven to have an almost 100% transmission rate. Therefore, a simple HybridNet was built by placing *srcMN* in Room #1, GW in Room #4, and 7 SNs in the hallway, as shown in Figure 8, where the hop between any two adjacent SNs has approximately 6 m, except for the first hop (*srcMN* to the first SN) and the last hop (the last SN to GW) that have a little shorter or longer distance. Note that four Wi-Fi access points (APs) have already been installed in the hallway and inside the rooms, so transmission interference may occur. As shown in Figure 9, the hybrid GW and hybrid node include a LoRa module and a WSN module connected via RS-232.

The experiment for different hops was conducted by moving the GW. That is, the GW was moved one hop at a time from the fifth location (four hops away) to the last ninth location (eight hops away), and experiments were conducted using five different images at each location, resulting in a total of 25 experimental runs.

For performance evaluation, three performance metrics were used: the packet delivery ratio (PDR), defined as the ratio of packets received at GW to all packets transmitted by *srcMN*; the image delivery ratio (IDR), defined as the ratio of images delivered without missing any packet to all images transmitted by *srcMN*; and *imgE2ED*. Table 5 shows key parameters and values used for experiments.

The proposed HMT protocol is compared with the PCT protocol [12], which has been published recently in terms of the above three metrics. Experiments were performed using 100 × 80 pixel RGB images captured with a small camera. Note that this RGB image is theoretically 24 KB in size, but in reality, the captured image is much larger as the color depth depends on factors such as the resolution and sharpness of the used camera. The captured image is compressed using JPEG2000 compression to a variable size of 1.65 KB to 2.5 KB, which is larger than expected, depending on the shape and content of the image [32]. Then, one image generates 17 to 25 image packets with a payload of 100 bytes, corresponding to an average of 21 packets.

#### 4.2.2. Transmission Interval

**Figure 10:**
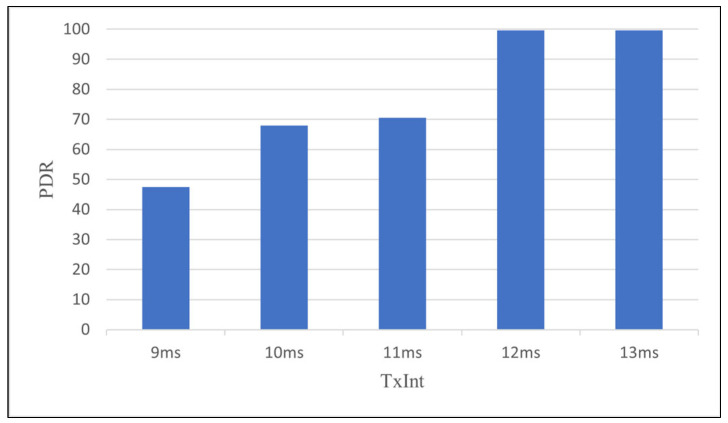
Packet delivery rate according to the changes of TxInt.

According to (9), *TxInt* was calculated to be approximately 11.27 ms. To verify the calculated value of *TxInt*, experiments were conducted using a small six-hop testbed, varying *TxtInt* from 9 ms to 13 ms in an indoor environment with minimal external interference. As shown in Figure 10, PDR dropped significantly at *TxInt* less than or equal to 11 ms; on the other hand, when TxInt is set to 12 ms or more, it is seen that PDR reaches almost 100%, supporting the correctness of the calculated value. Thus, for the rest of the experiments, *TxInt* is set to 12 ms.

#### 4.2.3. Image Delivery Rate

**Figure 11:**
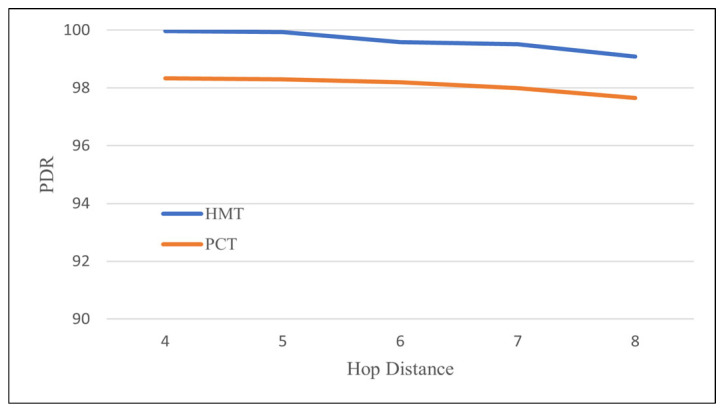
Packet delivery rate according to the increase in the hop distance of scrMN to GW.

Figure 11 compares the PDRs of the HMT and PCT protocols with increasing hop distance from *srcMN* to GW. Both protocols achieve high PDRs of over 97%, but they show a slightly decreasing pattern of PDR as the hop distance increases. Overall, HMT achieves about 1.8% higher PDR than PCT and shows a PDR of over 99% even at a fairly long hop distance of 8. This means that, at most, 1 packet can be missed when transmitting one image with an average of 21 packets. In general, data transmission in WSNs does not have a high PDR of more than 90% due to collisions between data transmissions within the WSN or collisions between data and control messages. In contrast, in HybridNet, it is judged that the WSN maintains a high PDR because transmission of other data or control messages within the WSN is completely excluded during image transmission.

**Figure 12:**
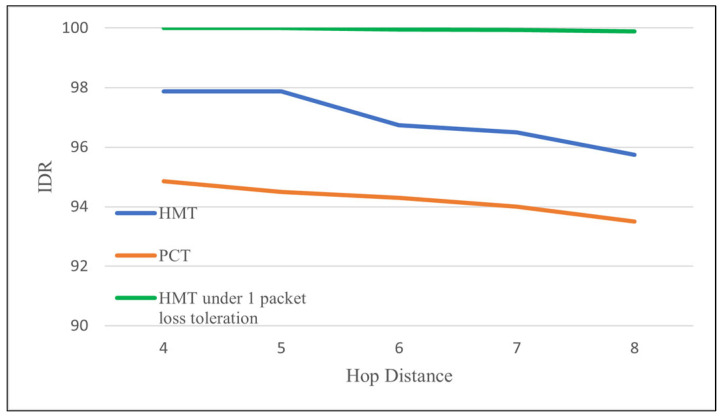
Image delivery rate according to the increase in the hop distance of srcMN to GW.

Figure 12 shows three graphs for IDR of the PCT and HMT protocols. The two lower IDR graphs, *PCT* and *HMT*, show PDR when an image transmission is considered a failure if any packet is lost during image transmission, while the upper HMT graph, *HMT under 1 packet loss tolerance*, shows IDR when image transmission is considered a success even if one packet is lost. In fact, looking at the images in the paper [32], it is shown that the quality of the image received under one packet loss is almost indistinguishable from that of a lossless image. Overall, HMT achieves about 2.3–3% higher IDR than PCT, but for long hop distances of 8 or more, its IDR drops below about 96%. However, if only one packet loss is tolerated, the IDR reaches almost 100%.

Experiments have demonstrated that HMT can achieve high PDR and IDR in image transmission using WSNs by completely separating scalar data transmission and image transmission in HybridNet but allowing LoRaNet to provide only minimal control message services such as image transmission start time and WSN path. Additionally, if it is assumed that the transmission distance between sensor nodes in a typical industrial environment is about 20 m, it can be expected that HMT can stably and quickly transmit images up to a distance of 160 m or more.

#### 4.2.4. Image Transmission Delay

**Figure 13:**
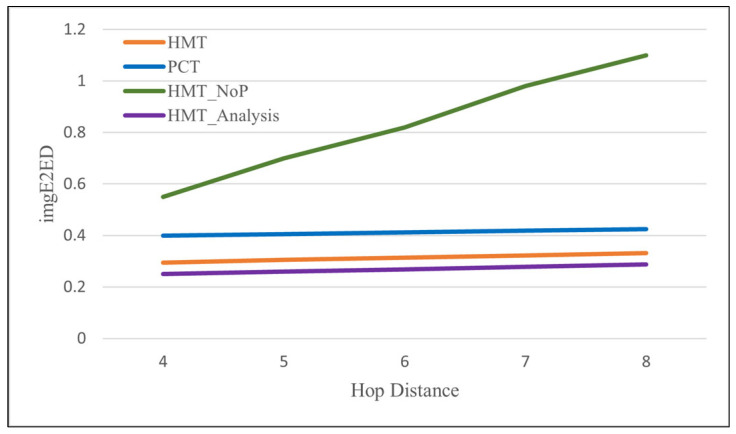
Image end-to-end delay according to the increase in the hop distance of srcMN to GW.

Figure 13 compares two protocols, HMT and PCT, that use pipelined transmission in terms of *imgE2ED* and also compares them with HMT_NoP, which does not use pipeline transport. The *imgE2ED* of HMT is about 0.3 s overall, but it shows a very small increase as the hop distance increases. This is because the time it takes for the first image packet to reach GW increases slightly. This means that *imgE2ED* is almost independent of the hop distance. It is also shown that the HMT graph matches well with the HMT_Analysis graph obtained with the values calculated according to (17); however, HMT_Analysis is positioned slightly lower because *TxInt* in the experiment was set to 12 ms, which is slightly higher than 11.27 ms in (9). Meanwhile, PCT shows *imgE2ED* of about 0.4 s overall. This is because PCT uses a larger transmission slot as it uses control messages to eliminate retransmissions and improve transmission reliability, but it shows a similar increasing pattern to HMT as it does not use retransmissions. As a result, HMT improves *imgE2ED* by about 25% compared to PCT. In contrast, for HMT_NoP, which does not use pipelined transmission, it is natural that *imgE2ED* increases almost linearly with the hop distance.

## 5. Conclusions

In this paper, we proposed an HMT protocol in which two networks with different transmission characteristics cooperate for the transmission of both periodic scalar data and on-demand images. With the help of the LoRa network, HMT is not only free from chronic network overhead problems caused by time synchronization, slot scheduling, and path setting in WSN for pipelined image transmission but also allows multimedia nodes to transmit images reliably and quickly without worrying about data and message collisions. It was demonstrated that the HMT protocol could not only transmit scalar data reliably using LoRaNet but could also reliably and quickly transmit an image of 24 KB in about 0.3 s using WSN.

## Figures and Tables

**Table 1:** Notations.

Notations	Description
*srcMN*	A multimedia node that transmits an image after receiving a command (*CMD*) from GW.
*WSNpath*	A sequence of nodes on WSN from *srcMN* to GW along which an image is delivered.
*dCH*	LoRa channel commonly used by all nodes to transmit commands or scalar data.
*dCHm*	LoRa channel only used for communication between *srcMN* and GW during image transmission.
$T_{h}$	Transmission time taken to transmit a packet to one-hop neighbor on WSN.
*TxInt*	The interval at which *srcMN* transmits image packets. In pipelined transmission, $TxInt=2\times T_{h}$.
*MaxRTs*	The maximum number of path-wide retransmission requests to salvage missing packets. In this paper, *MaxRTs* = 1.
*NS*[*x*]	A set of node *x*’s neighbors that indicates the link state of node *x*.

**Table 2:** Message notations.

Messages	Description
*DLm*	GW broadcasts a *downlink message*, *DLm* = ([*IREQ*]) at the beginning of each frame period, where *IREQ* = (*srcMN*, *WSNpath*, *WSNChList*, *dCHm*) where *WSNChList* is the list of WSN channels for pipelined image transmission.
*UL* *m*	Every node *x* sends an *uplink message*, *ULm* = (*x*, *scalarData*, [*NS*[*x*]]) every frame period, where *NS*[*x*] is required by GW to manage the WSN topology and is included only when the link state of node *x* is updated.
*Hello*	Upon receiving *DLm*, a node wakes up its WSN module every *k* frame period, where *k* is an integer, and broadcasts *Hello* = (ID) on WSN after a random delay time.
*iPacket*	An image packet, *iPacket* = (*seqNo*, *imgData*) where *seqNo* indicates a sequential number and *imgData* is a sliced portion of the image to be sent.
*RtREQ*	GW broadcasts a *retransmission request message*, *RtREQ* = $\left(m_{1},m_{2},\ldots ,m_{i}\right)$ where $m_{i}$, *i* = 1…*k*, is the sequence number of a missing packet.

**Table 3:** Parameters and values used in RT-LoRa evaluation.

Parameter	Value	Parameter	Value
Number of nodes	15	Spreading factors	SF7
Packet size	33 bytes	Bandwidth	125 KHz
Number of channels	1	Code rate	4/5
Tx Power	14 dBm	Preamble	8 symbols

**Table 4:** HW specifications used in the testbed.

Device	Specifications
GW mainboard	STM32MP151 [27] integrated with 32-bit ARM Cortex-A7: - Architecture: ARMv7-A - 32 Kbyte unified level 2 cache - MCU core frequency: up to 209 MHz
GW LoRa communication module	Semtech SX1308 [28]: - Sensitivity: up to −139 dBm - IO power supply to VSS: −0.5 V to 4.0 V - IF frequency: 125 kHz
LoRa device	Semtech SX1276 [29]: - Programmable bitrate: up to 300 kbps - Maximum link budget: 168 dB
WSN device	CC2630 Simplelink chipset [30] integrated with ARM Cortex M3 and CC2420 radio chip [31]: - Data rate: 250 kbps - Startup time: 1.015 ms - CCA time: 128 μs - PPD time: 192 μs - Flash memory: 128 Kbytes Supply voltage range: 1.8 V–3.8 V 2.4 GHz RF transceiver compatible with IEEE 802.15.4 PHY and MAC

**Table 5:** Parameters and values for HMT.

Parameter	Value
Image size (in Kbytes)	1.65–2.5
Payload (in bytes)	100
#packets per image on average	21
Number of WSN channels	3 (11, 14, 26)
Number of LoRa channels	2 (*dCH*, *dCHm*)
Tx power of WSN node	−12 dBm (≈10 m)

## Data Availability

Data are contained within the article.
